# Biophysical and Clinical Research on Acupuncture and Moxibustion

**DOI:** 10.1155/2015/518138

**Published:** 2015-01-26

**Authors:** Xueyong Shen, Lixing Lao, Yan Zhang, Ke Cheng

**Affiliations:** ^1^College of Acupuncture and Moxibustion, Shanghai University of Traditional Chinese Medicine, Shanghai 201203, China; ^2^Shanghai Research Center of Acupuncture & Meridians, Shanghai 201203, China; ^3^School of Chinese Medicine, The University of Hong Kong, Pokfulam, Hong Kong; ^4^Department of Family and Community Medicine, Laura W. Bush Institute for Women's Health, Texas Tech University Health Sciences Center, Lubbock, TX 79430, USA

Acupuncture has been used for thousands of years in treating diseases. Meridians and acupoints, on which acupuncture is practiced, have also attracted many research interests. Moxibustion is often used in conjunction with acupuncture as a thermal stimulation on acupoints. Laser, which generates extremely pure light of single wavelengths, is now used widely to stimulate acupoints, because it is noninvasive and easy to manipulate.

This special issue contains 1 human research investigating the electrical characteristics of the acupoints, 2 randomized controlled trials (RCTs) evaluating the effect of traditional moxibustion on patients with knee osteoarthritis and lumber disc herniation, respectively, 3 animal studies exploring the effect and mechanism of the traditional moxibustion and laser moxibustion on irritable bowel syndrome (IBS), cyclophosphamide-induced leucopenia, and knee osteoarthritis (OA), respectively, 1 systematic review reviewing the safety of moxibustion, 1 paper describing the design of an electrical thermal stimulation system comparable to moxibustion, and 2 animal researches studying the effect and mechanism of electroacupuncture on disc degeneration and hepatic blood perfusion respectively.

The results of these researches showed that acupoints may have special electrical characteristics at pathological status; moxibustion may relieve back pain and improve quality of life in patients with knee OA. Although the two RCTs did not report severe adverse events related to moxibustion, the systematic review found many factors may affect the safety of the moxibustion. The results of these researches also enhanced our knowledge of how traditional moxibustion, laser acupuncture-moxibustion, and electrical-acupuncture work in treating IBS, knee OA, cyclophosphamide-induced leucopenia, disc degeneration, and hepatic blood perfusion.

The encouraging findings in this special issue will promote further investigations in the fields of biophysical and clinical research of acupuncture and moxibustion and may encourage more use of moxibustion in clinic. The electrical thermal moxibustion and laser moxibustion might be good substitutes for traditional moxibustion, because they produce thermal effect but with no chocking smoke and smell, thus being more suitable in modern medical environment. However, in this current special issue, researchers only investigated the effect of laser moxibustion on animal and introduced a design of electrical thermal moxibustion device; further clinical research on both moxibustion devices is needed in the future.

